# African Swine Fever Survey in a European Context

**DOI:** 10.3390/pathogens11020137

**Published:** 2022-01-23

**Authors:** Ana de la Torre, Jaime Bosch, José Manuel Sánchez-Vizcaíno, Satoshi Ito, Carolina Muñoz, Irene Iglesias, Marta Martínez-Avilés

**Affiliations:** 1 Infectious Diseases and Global Health Department, Centro de Investigación en Sanidad Animal (CISA), Instituto Nacional de Investigación y Tecnología Agraria y Alimentaria-Consejo Superior de Investigaciones Científicas (INIA-CSIC), 28130 Madrid, Spain; torre@inia.es (A.d.l.T.); iglesias@inia.es (I.I.); 2 Animal Health Department, Centro de Vigilancia Sanitaria Veterinaria (VISAVET), Complutense University of Madrid, 28040 Madrid, Spain; jbosch@ucm.es (J.B.); jmvizcaino@ucm.es (J.M.S.-V.); satoshito@ucm.es (S.I.); caromuno@ucm.es (C.M.)

**Keywords:** pigs, wild boar, expert opinion, questionnaire, stakeholders, epidemiology

## Abstract

African swine fever (ASF) is currently the most threatening disease for domestic and wild pigs worldwide. Wild boar has been the main affected species in all EU countries except for Romania, where most notifications occur in domestic pigs. The spread of ASF in wild boar is challenging to control; risk factors are harder to identify and establish than in domestic pigs, which, together with an underestimation of the disease and the lack of treatment or an effective vaccine, are hindering control and eradication efforts. We distributed two online questionnaires, one for domestic pigs and one for wild boar, to experts of different background and countries in Europe, to explore risk factors in relation to ASF control connected to farming, hunting, trade, the environment, and domestic pig and wild boar populations. Overall, wild boar movements were estimated to pose the highest risk of ASF introduction and spread. The movement of pork and pork products for own consumption also ranked high. Here we explored, in addition to the assessment of risk pathways, the identification of risks of transmission at the domestic/wild boar interface, the importance of biosecurity practices and improved control efforts, and controversial opinions that require further attention.

## 1. Introduction

The unprecedented worldwide spread of ASF since it reached Europe in 2007, Asia in 2018, and now the Americas in 2021 turns ASF in the worst livestock pandemic of this century. The European Union (EU) was free from African swine fever (ASF), except for the Italian island of Sardinia, until 2014, when it entered through the Baltic countries and Poland [1,2]. Excluding Sardinia, from 2014 up to December 2020, there have been 6037 ASF outbreaks in domestic pigs in the EU and 39,970 ASF notifications in wild boar across 12 EU countries (Belgium, Bulgaria, Czech Republic, Estonia, Germany, Greece, Hungary, Latvia, Lithuania, Poland, Romania, and Slovakia) [3]. Only Belgium and the Czech Republic, both with only wild boar affected, have regained an ASF-free status [4,5].

**Figure 1:**
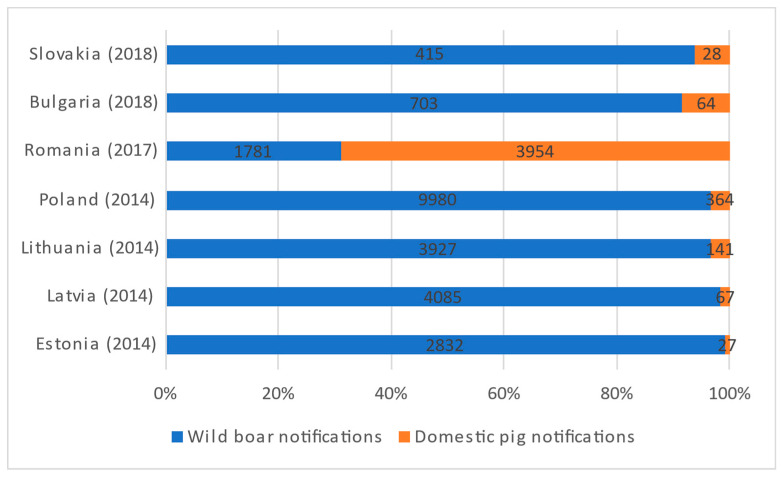
ASF notifications from 2014 to 2020 in countries with domestic pigs affected in the EU (except Sardinia), based on ADIS data. Year of first notification in brackets after country name.

**Figure 2:**
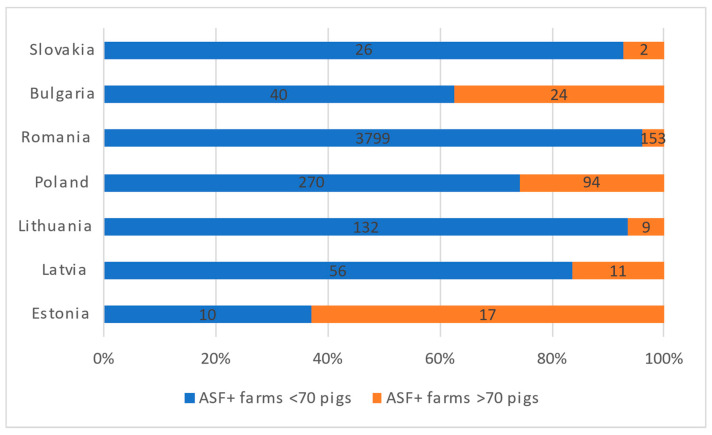
ASF notifications by farm size from 2014 to 2020 in countries with domestic pigs affected in the EU (except Sardinia), based on ADIS data.

Wild boar has therefore been the main affected species in all EU countries except for Romania, where most notifications occur in domestic pigs (Figure 1). Romania is also the EU country with the largest number of pig farms, more than 2 million in 2015, but up to 99% of these farms held 10 pigs or fewer [6]. Other affected countries such as Lithuania, Slovakia, or Bulgaria also have a similar average farm size. These figures are reflected in the number of outbreaks per country by farm size (Figure 2).

The risk factors for ASF spread in domestic pigs are well-known and have been reviewed within the EU recently [7,8]. Risk factors associated with ASF spread and persistence in wild boar are harder to identify and control. Through epidemiological analyses and modelling approaches, potential explanations of probable disease distribution have been explored [9,10]. As a wildlife species, exposure to disease depends on environmental risk factors (season, climate, land cover, connectivity between landscapes), human activities related with wild boar (population management, hunting, and gaps in biosecurity), human activities related with the environment (land use, farming, leisure, waste control), and wild boar ecology and behavior [11,12,13,14,15,16,17]. Given the high ASF incidence in wild boar, the domestic–wildlife interactions remain poorly assessed [18], and ASF remains underestimated. ASF virus has been circulating in wild boar in Europe for more than a decade, during which there has been an increase in the detection of asymptomatic wild boar positive for antibodies against ASF but negative for virus detection, thus immune to infection [19]. However, the number of immune wild boar remains low, and the infection keeps spreading.

So far, the main tools to control and eradicate ASF remain early detection; depopulation; contact tracing; and the establishment of movement restrictions, disinfection, and active surveillance within a protection and surveillance zone around each outbreak [20]. Depopulation, active carcass search and removal, the definition of an infected zone and surrounding surveillance zones, and the use of fences have also been recommended within the EU for wild boar [21]. The continuous spread of and difficulty in controlling ASF with the current tools have boosted the need to develop effective vaccines against ASF. The most promising protective vaccines against heterologous ASF strains are naturally attenuated virus [22], but to avoid infection with circulating virus, biosecurity measures should be taken. Furthermore, for effective control against ASF, it is essential that stakeholders involved in it are aware and have good knowledge and understanding about risk factors for ASF spread. Through the use of questionnaires, we aim to obtain an updated perspective on ASF risk of spread and control from experts of different countries and backgrounds that could represent the main stakeholders involved in ASF control, as well as to increase the awareness and engagement of stakeholders potentially involved in ASF control, in both wild boar and domestic pigs.

## 2. Results

### 2.1. Response Rate and Background

**Figure 3:**
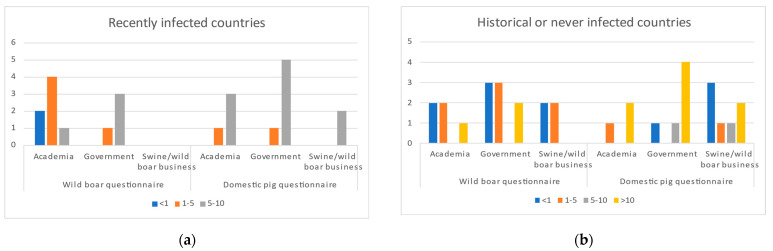
Summary results of years of ASF expertise by experts in each questionnaire. (**a**) Experts from Czech Republic, Estonia, Germany, Hungary, Latvia, Lithuania, and Russia; (**b**) Experts from France, Italy, Luxembourg, Portugal, Slovenia, Spain, and Switzerland.

The questionnaires were sent to 144 experts (72 wild boar experts and 72 domestic pig experts) from 24 different European countries, including experts from all ASF-affected countries in Europe. Experts from 14 countries (Figure 3) contributed to 56 questionnaires eligible for analysis (28 domestic pig and 28 wild boar questionnaires). The number of experts that replied to each question ranged from 22 to 28. Response rate, thus, varied between 31 and 39%. Twenty-four respondents finished all 52 questions in the domestic pig questionnaire, and 23 respondents completed the full 48 questions of the wild boar questionnaire. The domestic pig questionnaire was answered mostly by government veterinarians (43%), followed by those working in the private sector (32%) and in academia (25%). The wild boar questionnaire was mostly answered by experts working in government and academia, with background mainly in ecology but also in epidemiology, wildlife diseases, and hunting, and only by 4 experts from the private sector. Experts from historically or never-infected countries and domestic pig experts had more years of experience with ASF than recently infected countries and wild boar experts (Figure 3).

### 2.2. Pig Farming

A summary of the replies to questions on pig farming and our interpretation is provided in Table 1.

### 2.3. Wild Boar Population and Hunting Questions

A summary of the replies to questions on wild boar and hunting and our interpretation is provided in Table 2.

### 2.4. Wild Boar—Domestic Pig Potential Interaction

A summary of the replies to questions on wild boar–domestic pig interface and our interpretation is provided in Table 3.

### 2.5. Biological Vectors

A summary of the replies to questions on biological vectors of ASF and our interpretation is provided in Table 4.

### 2.6. Host Association Interactions

#### 2.6.1. Domestic Pig Questionnaire

**Figure 4:**
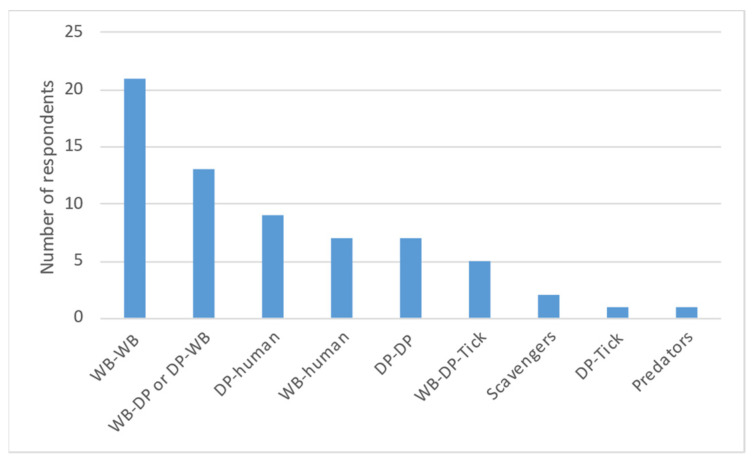
Summary results of most likely host association interaction for domestic pig questionnaire experts (WB = wild boar; DP = domestic pig).

28 experts replied to this question. Wild boar–wild boar was the most voted interaction (21 votes), followed by wild boar–domestic pig or domestic pig–wild boar (13 votes) and domestic pig–human (9 votes). Wild boar–human and domestic pig–domestic pig obtained seven votes each, wild boar–domestic pig–tick five votes, and domestic pig-tick only one vote (Figure 4). Experts of ES, IT and PT were the only ones to mention tick involvement (historical situation).

#### 2.6.2. Wild Boar Questionnaire

14 experts (56%, *n* = 25) considered it somewhat likely, likely, or very likely for wild boar and domestic pigs to come into contact. For 60% (*n* = 25), the most likely interaction between domestic pigs and wild boar would be indirect.

INTERPRETATION: It seems that domestic pig experts perceive the interaction among wild boar as the main interaction responsible for ASF spread. Among wild boar experts, the percentage of agreement on the likely contact between domestic and wild boar was higher (56%) than among domestic pig experts (46%). Indirect contact was estimated to be more likely than direct contact (60%).

### 2.7. Risk of Entry and Exposure Questions

#### 2.7.1. Domestic Pig Entry Pathways 

Wild boar movements, meat for own consumption, and illegal trade obtained the highest scores, with medium variability and medium-to-low uncertainty (Table 5). Catering waste was ranked with very low risk, with high variability and uncertainty. Indeed, it was one of the options together with the three mentioned considered as high-risk by experts that only ranked three or fewer introduction pathways (all from recently infected countries and IT).

For experts of recently infected countries, ranking was more homogeneous (Kendall’s W = 0.487, statistically a strong agreement), and the pathway with the highest risk was by far the introduction of ASF through wild boar movements. In contrast, for respondents from historically or never-infected countries, the introduction through wild boar movements ranked fourth, and the entry pathway with the highest and most homogenous scores was the potential introduction of contaminated meat or products for own consumption, followed by illegal trade (but with higher variability and less agreement). By background, there was a moderate agreement among respondents from government (Kendall’s W = 0.277) and among academics (Kendall’s W = 0.286) and a weak agreement among respondents from industry (Kendall’s W = 0.177).

#### 2.7.2. Wild Boar Entry Pathways 

The introduction through wild boar movements and through illegal trade of meat and products was also ranked as high-risk in the wild boar questionnaire, both with low uncertainty, but in the case of wild boar movement, a high variability (Table 6). Here, the number of respondents that ranked all pathways was higher. Agreement by Kendall’s W altogether, among the responses from experts from recently infected countries, and among historically or never-infected countries was weak. By background, only those from government and from academia replied to the risk assessment questions, and only respondents from government achieved a moderate agreement (Kendall’s W = 0.265). More than 50% of wild boar experts estimated it was extremely probable that ASF was introduced or reintroduced from infected neighboring countries through wild boar natural corridors or patches of habitat shared between borders, and a further 30% estimated it was somewhat probable.

#### 2.7.3. Domestic Pig Exposure Pathways

The exposure pathway with the highest risk was indirect contact between domestic pigs and wild boar (low variability and uncertainty). Other high-risk options considered by domestic pig experts included direct contact between domestic pigs and wild boar and vectors, but either variability or uncertainty were high (Table 7).

The variability in ranking for the direct contact between domestic pigs and wild boar pathway was so high among domestic pig experts of recently infected countries that there was not a most-agreed-upon score, while for domestic pig respondents from historically or never-infected countries, it was ranked as medium risk. Spread through mechanical vectors was also ranked differently in the domestic pig questionnaire: medium to low by experts from historically or never-infected countries and medium to high by experts of recently infected countries. Kendall’s W revealed a moderate agreement among domestic pig exposure pathways responses from recently infected countries (0.305) and strong agreement among government (0.383) and academy (0.445) respondents of all countries.

#### 2.7.4. Wild Boar Exposure Pathways

For wild boar experts, the exposure pathways with the highest risks were direct and indirect contact between wild boars (medium variability, low uncertainty) (Table 8). Direct contact between wild boar and domestic pig was also ranked as medium-risk (low variability and uncertainty) among wild boar experts. In the wild boar questionnaire, there was only a moderate agreement among respondents of historically or never infected countries (Kendall’s W = 0.315). By background, wild boar government respondents strongly agreed (Kendall’s W = 0.421), while the agreement among wild boar academics was weak.

### 2.8. ASF Prevention and Control and Socio-Economic Questions

A summary of the replies to questions on ASF control measures and our interpretation is provided in Table 9.

## 3. Discussion

The objective of this expert elicitation was to obtain information and increase awareness on risk factors for ASF spread and control in Europe. The experts were mainly veterinarians and ecologists from administration and research involved in disease control, as well as from the swine and hunting industry. We aimed at reaching those already involved or that could potentially be involved in ASF control. Some experts replied to both the domestic pig and the wild boar questionnaires, but the majority of experts replied to just one of them. We also aimed at having representation from infected and non-infected countries. However, the number of respondents per country was not homogeneous, with Italy, Latvia, Portugal, and Spain being the countries with the highest number of respondents. By background, only private industry (wild boar hunting/domestic pig) was less represented in the wild boar questionnaire, particularly in the risk assessment questions. The perception by hunters of ASF control has been assessed in Estonia, Latvia, Lithuania, Bulgaria, Germany, and Russia [23,24,25,26]. Our study complements the perception of ASF risk factors and control by other stakeholders, such as people engaged in wildlife management and research as well as those familiar with both domestic pigs and hunting, and increases the study area to other parts of Europe as well.

The starting point to recruit experts was contacting partners from the EU project on ASF vaccines and vaccination, VACDIVA. While this ensured a relevant expertise in ASF, it may have also influenced the higher number of experts from the countries mentioned, all of which are represented in the project. The main limitation of the questionnaire design was it was too lengthy, taking at least 30 min to complete. We did aim to reduce as much as possible the content and split the original questionnaire in two, aiming to gather opinions from both domestic pig and wild boar experts. Experts were warned in advance of the time of completion, but around 5 experts out of 28 were lost towards the last questions. This time could have also discouraged experts and influenced the final number of respondents. While the response rate seems low (30%), 91% of the experts who replied were either from the VACDIVA Consortium or recommended by a partner from VACDIVA. Therefore, the opinions, while not a comprehensive sample that represents a whole sector or country, allow for analyzing perceptions and information about important factors in ASF control, and given the variability of cultures, environmental conditions, and even domestic pigs and wild boar exploitation within the EU, the authors considered it appropriate to summarize and compare results per country or group of countries as well as per background.

This study has provided an updated assessment of the main perceived risk pathways of ASF entry and spread in infected and historically or never-infected countries. In this way, the main risk perceived for both the entry and spread of ASF is associated with wild boar. This was particularly evident for recently infected countries, which are clearly very aware of the continuous risk of re-introduction of infected wild boar from neighboring countries with ASF. Several authors [27,28] already indicated that the existence of suitable wild boar habitats at the borders increased the risk of ASF entry in these countries. The entry pathway with the second-highest risk of introduction for recently infected countries and highest for historically or never-infected countries was estimated to be the illegal movement of products and the introduction of contaminated products for own consumption. The pathways ranked with lowest risk were the legal movement of pigs, pig products, and fomites. Assuming that the EU is in a “high-risk period” because of the high threat of ASF at the moment, our results align with Mur al. [29], who considered wild boar movements as one of the main entry pathways. The low-risk estimation of legal movement of pigs could similarly be attributed to the increased awareness because of the “high-risk period” situation. However, illegal movements are today ranked with a higher risk.

Swine production across the EU is diverse, and several pig farming systems coexist, with industrialized farming, traditional small-scale farming, and specialized farming (local breeds or organic) being the most common [30]. In many countries of Eastern Europe, the traditional small-scale or backyard farming is predominant, but in Western Europe, the predominant form is industrialized farming, including specialized extensive production such as in the Iberian Peninsula that should not be compared with non-commercial outdoor pig farming. Swill feeding is banned across the EU [31] but was admitted to still happen, although not frequently, by the respondents of the domestic pig questionnaire. In fact, the biosecurity measures against ASF that are estimated to be complied with by a smaller number of non-commercial establishments according to the results of the domestic pig questionnaire included feed and water control, cleaning and disinfection of structure and equipment, animal health education, measures for visitors and farm workers, and pest control. Animal health education or limited farm visits with proper register and protective equipment had also been assessed as very important in Jurado et al. [8]. However, even if most of the ASF notifications in domestic pigs have occurred in the backyard sector, the risk of ASF occurrence can also affect the rest of pig farming systems.

Given the high apparent incidence of ASF in wild boar in Europe, direct and indirect contact with wild boar appear as important risk factors for ASF spread to domestic pigs. Some important risk factors identified through the questionnaires were the location of farms in habitats highly favorable for wild boar, the existence of hybrid pigs, domestic pig farmers who also practice wild boar hunting, and the access of wild boar to crops around domestic pig farms. Despite these interactions, contact between domestic pigs and wild boar was ranked as medium to low risk by both domestic pigs and wild boar experts. However, when compared with other exposure pathways, the indirect contact between domestic pigs and wild boar was ranked the highest for domestic pig experts. The indirect interaction between wild boar and domestic pigs has been suggested as the most likely source of ASF transmission in several field studies [32,33,34,35]. For wild boar experts, the most likely interaction between domestic pigs and wild boar would also be through indirect contact, but the highest risk exposure pathways were attributed to wild boar–wild boar interactions. The practices that could spread the virus from domestic pig establishments to the surrounding environment were estimated to happen less frequently than access of potentially infected wild boar to the surroundings of a pig establishment. However, recycling manure as an organic fertilizer in crops is a common practice in the EU. A survey of farmers in France on manure management indicated that 36.5% of manure is spread on grassland, 39.6% on maize ground, 12.9% on cereal land, and 7.9% on oilseed/protein crops [36].

The practices that could spread the virus from wild boar to domestic pig establishments include gathering bedding and other materials from the environment. Access of wild boar to crops around farms was estimated to happen frequently, particularly in summer but also in autumn and spring, with winter being the season with lowest probability of crop damage by wild boar. For reasons yet to be elucidated, ASF in Europe seems to peak in autumn in wild boar and in summer in domestic pigs [12,37]. Crops mentioned by experts in this questionnaire, which grow in summer and are accessed by wild boar, included cereals (mainly maize and oat), protein crops (sunflower), and potato, which offer food and shelter to the animals. Indeed, the highest percentage of ASF notifications have occurred in Europe in agroforestry landscape (including monoculture areas). Agriculture accounts for around 35% of the total land within the EU [38] and is estimated to expand because of the projected increase in the demand for agricultural commodities (70–100% by 2050) [39]. Specifically, the use of land for cereals, protein crops, and fodder for animal feed, human consumption, and industrial purposes is expected to grow in the EU. The European Commission has estimated a significant growth for total EU cereal production (mainly wheat and maize) (up to 319 million t by 2030) and feed crops such as oat (260 million t in the medium term) [40]. The relationship between virus spread and the seasonal ASF summer peak in domestic pigs, where high environmental contamination in the surroundings of pig establishments seems to exist due to the available feed and shelter offered by crops to potentially infected wild boar, should be further studied. In addition, flying insects carrying potentially infectious blood are also present in larger amounts in the warmest months, having a potential impact particularly on local short-distance spread. Interestingly, ASF notifications in the EU in agroforestry landscape have increased by 30% in wild boar and by 80% in domestic pigs since 2016 (based on notifications to the Animal Disease Information System of the EU). Between 2007 and 2016, ASF wild boar notifications were more prevalent in natural areas in the EU (78.5%), followed by agroforestry areas (21.3%) and agro-urban areas (0.5%). In contrast, in non-EU countries (Russian Federation, Belarus, Ukraine, and Caucasus region), the distribution of ASF notifications was 57.5% in natural areas, 40.9% in agroforestry areas, and 1.6% in agro-urban areas (1.6%). In domestic pigs, ASF occurred mainly in natural areas in the EU (63.6%), in contrast with 20.5% of notifications in natural areas in non-EU countries. Oppositely, in non-EU countries, the main landscapes for ASF notifications in domestic pigs were agroforestry areas (42.3%) while in the EU, they accounted for 16.4% of notifications. In any case, wild boar movement was the entry pathway ranked with the highest risk by experts of both domestic pig and wild boar groups. The introduction of ASF through infected pork or pork products for own consumption and through illegal trade was also ranked with a high-to-very-high risk. However, it was surprising to find the introduction pathway through catering waste being ranked with a low risk, considering that this has been a major suspected pathway of introduction of transcontinental ASF spread as well as origin of recent outbreaks [41,42].

A few questions obtained contradictory responses, such as the role of scavengers and predators in the spread of ASF. The existing scientific literature also reveals inconclusive evidence. On one hand, scavengers reduce the time a carcass remains in the environment, decreasing environmental persistence, but on the other hand, not all carcass remains are removed, and bones in particular will remain [17,43]. ASF virus genome can remain preserved in bones and the remnants of bone marrow of buried carcasses for more than two years [44]. Furthermore, some mammals and birds can transport pieces of meat in their mouths or beaks, contributing to local ASF spread [43]. For the moment, we can only hypothesize that scavengers could contribute both to an increase and a decrease in ASF local spread and persistence risk. One should also bear in mind that wild boar could scavenge its own species’ remains [45], which is considered a high-risk factor for the spread of ASF that is minimized through effective carcass search and removal [21]. Scavenger species that were mentioned other than wild boar and pigs (domestic and feral) included foxes, small and large carnivores (martens, badgers, water vole, polecat, raccoon, dogs, golden jackal, and bear), birds (corvids and birds of prey), and insects. Depending on landscape, season, and time of the day, there will be different species scavenging on carcasses [46]. In open habitats of the European temperate woodland, ravens, common buzzards, white-tailed eagles, and domestic dogs were found to be the most common scavengers. In the forest, pine martens, jays, and wild boar predominated. When temperature decreased, scavenging increased, except for raccoon dogs. In the Mediterranean ecosystem, the most common scavenger in open habitats was the griffon vulture (during the day), while in vegetation-covered habitats, the most frequently mammal scavengers were red fox and, in the evenings and nights, wild boar. As for predators, there was a high agreement among the questionnaires’ respondents that the main predator animal species of wild boar is the wolf, although other species such as foxes and jackals can also predate on wild boar. Studies on food habits of wolves indicate that wild boar is their main prey, reaching up to 50% of wolves’ diet [47,48]. All experts indicated that predation occurred mainly on piglets or juveniles, being more frequently in spring, although they can also predate during the whole year, as 48% of experts agreed and in line with other studies [43,44]. Two new concerns expressed by experts were the possibility of wild boar increasing movements and range, leading to ASF dispersal because of wolf activities, and the potential overlapping of wolves and other potential predators with wild boar in natural and agroforestry areas. Therefore, even if the direct effect on ASF spread by wild boar predators is inconclusive, one could attribute an indirect risk of ASF dispersal by wild boar to the presence of predators in an area.

Regarding control measures, from the questionnaire replies we found that the existence of a shared surveillance program for ASF across neighboring countries of different geopolitical scope is more than necessary to focus control efforts in a coordinated manner. Evidently, vaccination in wild boar would also help control efforts, and there seems to be acceptation among those surveyed to support such a plan. However, despite vaccination, biosecurity measures would still be necessary to achieve an effective ASF control.

## 4. Materials and Methods

Two online questionnaires, one on domestic pigs and one on wild boar, were designed and developed using the Surveymonkey™ tool (Momentive Inc., San Mateo, CA, USA) to collect information and gather perspectives of stakeholders involved in ASF control across EU countries.

A review of the scientific literature was conducted to draft the questions on risk factors for ASF spread and control and to identify the main entry and exposure pathways in wild boar and in domestic pigs. Questions aimed at extracting at least basic information (y/n or multiple choice answers) but also tried to allow quantification or qualitative estimates of how common or frequent certain practices were as well as when and where they occurred more frequently. A pilot version was distributed among members of CISA-CSIC (National Animal Health Research Centre) and VISAVET-UCM (Veterinary Surveillance Research Centre at the Complutense University of Madrid) teams who were not previously involved in the questionnaire development, and questions were improved in format and clarity. Finally, the questionnaire was evaluated for comprehension by two experts, a wild boar veterinarian researcher and a domestic pig veterinarian practitioner, with more than 10 years of expertise in their areas of knowledge and in ASF.

The final version of the domestic pig survey included 21 questions on risk factors for ASF spread mainly linked to backyard farming, 20 questions about potential direct or indirect interactions between domestic pigs and wild boar, and 9 questions on ASF control and impact of ASF vaccination (Table S1). The final version of the wild boar survey included 11 questions on wild boar hunting, 14 questions linked to the potential direct or indirect interactions between wild boar and domestic pigs, 8 questions linked to wild boar predators and scavengers, and 13 questions about ASF control and vaccination impact. In each survey, experts were asked to rank several pathways for the potential introduction of and exposure to ASF in their countries (Table S2). Both surveys included seven additional questions on background information (personal details, place of work, background knowledge, main area of expertise and years of experience in it, years of experience with ASF, and country for which they would be filling the questionnaire). In addition, experts were given the opportunity to comment on their replies in relation to the risk of ASF through open questions to facilitate interpretation. Furthermore, for questions to which responding was compulsory to continue with the survey, the options “I don’t know”, “not applicable”, or “other” (free text) were included as necessary.

The experts were selected from different backgrounds (academia, government, and swine industry/practitioners or wild boar management and hunting) through nomination by partners of the EU H2020 Innovation Project VACDIVA [49] and by the experts who tested and assessed the pilot questionnaires. VACDIVA experts were also invited to participate. A total of 72 domestic pig experts and 72 wild boar experts were sent the questionnaires’ links and QR codes by email and asked to complete them between the 8th and 30th of April of 2021. Each expert was asked to specify the country for which they would be replying to the questionnaire. Experts could choose to reply to one or both questionnaires.

The answers were anonymized using a reference number assigned to each expert and transferred to an Excel spreadsheet linked to the country, background, and expertise of each respondent. A descriptive study was conducted on the experts’ backgrounds and opinions. Simple summaries per question were available in SurveyMonkey but were also analyzed in Excel by country or group of countries and background. Similarly, for the ranking questions, the level of agreement between respondents was evaluated by background (government, academy, or industry) and by country (recently infected vs. historically or never-infected) using Kendall’s W, which is a non-parametric measure of concordance between rates (W-value < 0.26 = weak; between 0.26–0.38 = moderate; >0.38 = strong agreement) [50]. Uncertainty was assessed by counting the number of respondents who ranked similarly (for >15 respondents: >8 = very low; 7–8 = low; 5–6 = medium; 4–3 = high; <2 = very high uncertainty; for <15 respondents: 6–5 = low; 4–3 = medium; <2 = high). Variability was assessed by dividing the scores into two groups, low (scores 1 to 4) and high (scores 5 to maximum), then adding up the subtotals per group and obtaining the absolute difference between groups. The value obtained was interpreted as follows: 0–1 = very high; 2–5 = high; 6–9 = medium; 10–13 = low; >14 = very low variability. Countries abbreviations are based on ISO country codes.

## Figures and Tables

**Figure 5:**
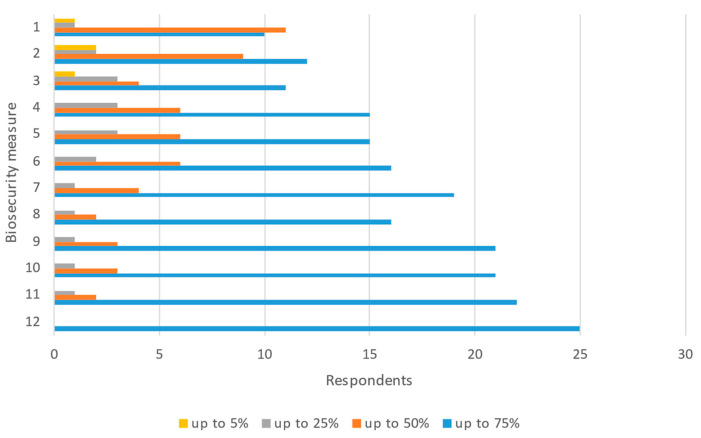
Percentage of commercial pig establishments estimated to comply with biosecurity measures for ASF prevention. 1: All-in-all-out or similar biosecurity system; 2: Pathways from clean to dirty signaled and easy to follow; 3: Quarantine room; 4: Pest control, including measures against mechanical vectors (flies, birds, rodents, etc.); 5: Cleaning and disinfection of vehicles’ wheels; 6: Perimetral fencing; 7: Preventive measures for visitors, farm workers, and vets (recording, protective equipment, and so on); 8: Cleaning and disinfection of structure and equipment; 9: Feed and water control; 10: Appropriate removal of carcasses, slaughter residues, manure, and food waste; 11: Daily check for clinical signs and mortality; 12: Identification of animals and farm records, including animal movements.

**Figure 6:**
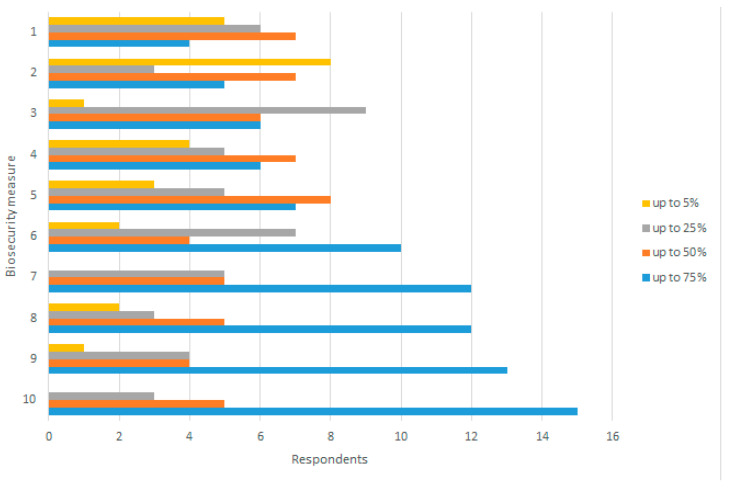
Percentage of non-commercial pig establishments estimated to comply with biosecurity measures for ASF prevention. 1: Pest control, including measures against mechanical vectors (flies, birds, rodents, etc.); 2: Preventive measures for visitors, farm workers and vets (recording, protective equipment,…); 3: Animal health education; 4: Feed and water control; 5: Cleaning and disinfection of structure and equipment, before and after home slaughtering; 6: Containment of pigs, not allowing contact with outside pigs, feral pigs, wild boar, or their products; 7: No sharing of equipment, feed or bedding materials between farms; 8: Appropriate removal of carcasses, slaughter residues, manure, and food waste; 9: No movements between/from non-commercial farms; 10: Access to animal health services.

**Table 1:** Summary results of domestic pig farming questions (% of agreement, *n* = number of experts).

Factors	Results
Backyard pig farming	Yes (97%, *n* = 20), in <5% total farms (44%, *n* = 26, EE, ES, FR, IT, LV, LT, PT). At least 50% or >50% of backyard farms (30%, *n* = 26, CZ, IT, LV, LT)
Outdoor pig production	Common in limited areas or with veterinary supervision and regulations (ES, FR, IT, PT)
Swill feeding	Yes (39%, *n* = 28) but in self-consumption farms, in specific areas (ES, IT), or all over the country (rest)
Home slaughtering	Likely or somewhat likely (57%, *n* = 28), for self-consumption. Somewhat unlikely or unlikely for 8/9 experts from the swine industry. Official vet present prior to slaughter (58%, *n* = 24)
Farming + hunting	Very likely, likely, or somewhat likely (78%, *n* = 25)
Unidentified movements	In backyard farms ≤ 3 pigs (EE, any time of year; LV, spring; IT, winter; in the whole country: EE, LV, PT; in specific regions: FR, IT). Local movements for 29% (*n* = 28), pig products from home slaughter given as gifts particularly at Christmas or during winter. Considered low-risk
Multiple sources of pigs	Considered low-risk
INTERPRETATION	Risk from backyard farming persists because of high-risk practices such as swill feeding, home-slaughtering, farming, and hunting or unidentified movements. No evidence of such risk practices happening in outdoor farming with veterinary supervision and compliance with regulations.

**Table 2:** Summary results of wild boar and hunting questions (% of agreement, *n* = number of experts).

Factors	Results
Wild boar abundance and distribution	Native species (93%, *n* = 28), widespread with high abundance (all except for LV and EE: widespread, low abundance; and local areas of ES, PT (patchy, high abundance). Increasing population trend in the last 5 years (21/28), except recently infected countries, although some have observed the population rising again. Mainly in forest (100%) and agroforestry areas (96%) (*n* = 28), followed by shrub lands or monoculture areas (82% each), urban areas (71%), and grass-lands (68%)
Hunting	Commonly hunted species, in winter (100%, *n* = 28) and autumn (93%, *n* = 28), when driven hunts or hunting with dogs is allowed. Occasionally hunted in spring and summer (50%), to prevent damages in fields or as single hunts. In LV, HU, hunting of wild boar throughout the year. In total, 20–30% (33%, *n* = 28) or 50% or above (33%, *n* = 28) of wild boar are hunted, generally by recreational hunters. Illegal hunting not common but inconclusive by country
INTERPRETATION	Risk from wild boar exists because of its abundance and distribution, including in agroforestry areas. Risk from hunting is higher in winter and autumn.

**Table 3:** Summary results of wild boar–domestic pig potential interaction questions.

Factors	Results
Direct interaction	Hybrid pigs exist (60%, *n* = 25), but unlikely occurrence (65%, *n* = 52), although likely, somewhat likely, or very likely in specific regions, i.e., in IT, PT, and ES where wild boar habitat overlapped with outdoor or extensive pig production. In some places in RU people intentionally hybridize domestic pigs with wild boar. If it was to happen, interaction with wild boar would be more likely in agroforestry areas (52%, *n* = 25), followed by forest (24%), shrub land (12%), and grasslands (12%). Risk estimation by experts varied by location, generally low. Only experts from ES and PT considered high risk as an option
Indirect interaction	Existence of pig farms in suitable wild boar habitats (100%, *n* = 25), >60% of farms in such habitats (54%, *n* = 26), although it varied by country from 100% (EE) to less than 20% (IT) Wild boar access crops around farms (96%, *n* = 27), particularly in summer (100%, *n* = 14) but also in autumn and spring, winter being the least likely season to have crop damage by wild boar (67%, *n* = 9). For 12 experts (CZ, EE, ES, FR, IT, LV, PT), all year round. For 8 experts (EE, IT, LV, LT) this did not happen in winter or spring. Maize or corn was the crop most frequently mentioned by experts as the most damaged by wild boar in their countries, followed by wheat, potato, and meadows. Potatoes, wheat, and oats were more frequently mentioned by experts of northern countries, while corn or sunflower was more cited by experts of more southern European countries Dumping manure/waste outside farm: yes (85%, *n* = 26), in >60% farms (36%, *n* = 22) and in <1% farms (32%, *n* = 22) Collecting bedding or forage from environment: <20% of farms or none (50%, *n* = 26) but % of farms increased if backyard or outdoor farming Human activities in wild boar areas: mushroom picking, wood harvesting, and timber cutting (>80% of agreement) Wild boar access to urban areas or areas with human garbage: common in Italy and Hungary; inconclusive for the rest. Replies from “not so common” to “common” depending on background Risk estimation: medium-low (69%, *n* = 26), lower in recently infected countries and higher in countries with outdoor production
INTERPRETATION	Historically or never infected countries had a higher risk estimation from wild boar–domestic interaction than recently infected countries, even if common access of wild boar to crops around farms or urban areas or even if farms located in wild boar suitable habitats.

**Table 4:** Summary results of biological vectors questions.

Factors	Results
*Ornithodoros*	Not found in current pandemic, so experts less aware unless historic infection. A third of the respondents (*n* = 54, wild boar, and domestic pig respondents together) admitted not knowing very much about *Ornithodoros* ticks
Other insects, rodents, birds	The domestic pig experts admitted that it is difficult to avoid the entrance in farms of rodents (mainly mice and rats), insects (particularly flies and mosquitoes), or birds (sparrows, ravens, scavengers in outdoor farms), although there are biosecurity measures to reduce their risk of entry. In addition, experts of infected countries, particularly EE and LV, stated that they never found an association or evidence of association between potential mechanical vectors and ASF. In contrast, in IT, an expert pointed at the suggestion of insects playing a limited but not negligible role in ASF spread between neighboring farms in April–June in Sardinia
Wild boar predators	Inconclusive opinion on the role of predators in ASF spread (30% contribution to spread, 30% reduction of spread, 20% no role, *n* = 26). Wolf, main predator (72%, *n* = 25). Other species mentioned were golden jackals (HU, SI), lynxes (LV), foxes, small carnivores, and birds of prey. Wild boar experts clarified that if predation occurred it would be mainly on piglets or juveniles, so more frequently in spring (also because of predation by other smaller species such as foxes or jackals), although 12/25 estimated wolves would predate during the whole year round. Wolves and other potential predators are also distributed in natural and agroforestry areas according to the experts, overlapping with wild boar
Wild boar scavengers	Inconclusive opinion on the role of scavengers (36% contribution to spread, 36% reduction of spread, *n* = 25). Fox, main scavenger (60%, *n* = 25). Other species mentioned were other small carnivores (martens, badger, water vole, polecat, raccoon), birds of the *Corvidae* family, birds of prey, wild boar, insects, wolves, golden jackal, dogs (domestic or stray), pigs (domestic or feral), and bear. Scavengers are thought to be present the whole year round in all types of landscapes but are more prevalent in natural and agroforestry areas
INTERPRETATION	More research and awareness is needed on the existence and role of vectors in ASF spread.

**Table 5:** Assessment of risk of ASF entry through different pathways in the domestic pig questionnaire.

Entry Pathways	Respondents	Most Agreed Score	Variability	Uncertainty
Wild boar movements	20	Very high risk	Medium	Low
Own consumption	19	High–very high risk	Medium	Low
Illegal trade meat/products	18	Medium-high risk	Medium	Medium
Illegal trade live pigs	17	High risk	Very high	High
Legal trade live pigs	15	Low risk	High	High
Legal trade meat/products	14	Medium risk	Low	Medium
Catering waste	15	Very low risk	High	High

**Table 6:** Assessment of risk of ASF entry through different pathways in the wild boar questionnaire.

Entry Pathways	Respondents	Most Agreed Score	Variability	Uncertainty
WB movement	24	Very high risk	High	Low
Illegal trade meat/products	24	High–very high risk	Very low	Low
Illegal trade live animals	23	Medium-high risk	Very high	Medium
Legal trade live animals	22	Medium-low risk	Medium	Medium
Legal trade meat/products	21	Medium risk	High	Medium
Catering waste	20	Low risk	Medium	High
Water sources	20	Very low risk	Low	Very low

**Table 7:** Assessment of risk of ASF exposure through different pathways in the domestic pig questionnaire.

Exposure Pathways	Respondents	Most Agreed Score	Variability	Uncertainty
WB—indirect contact	22	High–very high risk	Low	Low
DP—indirect contact	19	Medium risk	Very low	Medium
Waste feed	19	Medium risk	Very high	Medium
WB—direct contact	19	Medium-high risk	Medium	High
DP—direct contact	18	Not agreed	High	High
Vectors	17	Medium-high risk	High	Medium
Water	14	Medium-low risk	Medium	High

**Table 8:** Assessment of risk of ASF exposure through different pathways in the wild boar questionnaire.

Entry Pathways	Respondents	Most Agreed Score	Variability	Uncertainty
WB—indirect contact	22	High risk	Medium	Low
DP—indirect contact	23	Medium risk	High	Low
Waste feed	22	Medium risk	High	Medium
WB—direct contact	21	Very high risk	Medium	Very low
DP—direct contact	21	Medium risk	Low	Low
Vectors—ticks direct contact	18	Very low risk	Medium	Low
Water, rivers, ponds	21	Very low risk	Low	Medium-low

**Table 9:** Summary results of ASF control measures (% of agreement, *n* = number of experts).

Factors	Results
Contingency plans and surveillance	Stakeholders aware of contingency plans (69%, *n* = 26), but active surveillance based on sampling after a clinical suspicion (most voted option), or routine sampling based on census rather than risk of entry/exposure (only mentioned by 4 experts from ES and IT)
Biosecurity measures domestic pigs	Commercial establishments: most experts perceived compliance with biosecurity measures to be very high, with all measures mainly scoring above 75% compliance (Figure 5). A compliance of 50% was scored by 44% and 36% of experts (*n* = 25) for the measures “All-in-all-out or similar biosecurity system” and “Pathways from clean to dirty signaled and easy to follow”. A total of 60% (*n* = 25) considered that all the control measures proposed were complied with by more than 50% or 75% of commercial pig establishments Non-commercial establishments: more than 50% of domestic pig experts (median *n* = 22) estimated more than 75% compliance for the measures “access to animal health services”, “no movements between/from non-commercial farms”, “appropriate removal of carcasses, slaughter residues, manure and food waste” and “no sharing of equipment, feed, or bedding materials between farms” (Figure 6). The measures with lower compliance scores also registered a higher variability among experts, i.e., preventive measures for visitors, farm workers, and vets; pest control; feed and water control; or cleaning and disinfection of structure and equipment, before or after home slaughtering.
Wild boar control measures	PCR testing of dead wild boar most common control measure (87%, *n* = 23), followed by PCR testing of hunting wild boar and PCR and antibody testing of hunted wild boar (50%). The least voted was fencing (4/23) Main measure to control human involvement in spread: specific training for hunters and incentives for hunting (90%, *n* = 20). Other measures mentioned were the use of hunting dogs trained to find carcasses (for 5 experts) or banning human activities in a potential infected wild boar habitat, including crop areas, picnic areas, sports in nature, tourism in natural areas, or recreational hunting (for 8 experts) Signs prevent ASF (87%, *n* = 23) but not people’s access to infected areas (35%, *n* = 20). Other places mentioned by experts that were not included in the questionnaire were highway parking sites, administration buildings and web pages, local markets, buses, train stations, agricultural shops, and in the media. Shared surveillance plan: inconclusive results. Almost half of the wild boar experts (10/23) did not know about it. Eight estimated it existed and four that it did not, but experts from the same country provided opposite replies Hardest measure to implement: 21 experts replied, with a myriad of options (more than one possible): depopulation (33%); human behavior changes, such as stopping any activities related with wild boar in infected areas but also preventing the introduction of home-made pork products from infected countries by farm workers, which has failed in the past (29%); fencing; access to waste; hunting biosecurity, including during the finding and disposal of carcasses Baiting or supplementary feeding: only legal in autumn (85%) and winter (75%) (*n* = 15). For 10/25 (40%) respondents, supplementary feeding was an illegal practice, but baiting was allowed. Respondents from EE, ES, LV, and LU specified certain measures that were taken to reduce sanitary risks from baiting: use of limited amount of feed, automatic feeders, or forage dispensers, or restricting baiting to specific hunting areas or controlled extensive wild boar breeding farms Carcass removal: enforced (68%, *n* = 25), with no seasonal differences. Rendering plants for storage and destruction of potentially infected wild boar carcasses were available in all the countries except for experts from IT
Vaccination and economics	Most likely in wild boar (86%, *n* = 21), paid by government (87%, *n* = 23). Wild boar is either traded (52%) or valuable for hunting tourism (65%) (Both, *n* = 23). Sharing wild boar products is common (96%, *n* = 23). Moving live wild boar for hunting purposes is not allowed or not in place for 12/23 respondents, but wild boar are legally traded for hunting within the country for 7 respondents and illegally introduced or moved within the country for 5 experts from recently infected countries (CZ, HU, RU) Commercial farms’ vaccination costs were estimated to be covered by the industry by 50% of experts. Backyard farming pigs would not be vaccinated (EE, LV). Pig farmers would very likely or likely accept ASF vaccination as a measure to control the disease (76%, *n* = 25). Main perceived trade activity: CZ and IT, importer of pigs; EE, LV, PT, importer of pig products; ES, exporter of pig products
INTERPRETATION	There seems to be room for improvement for the following: wild boar surveillance, specific control measures at pig establishments, signaling to prevent people entering at-risk places. However, applying control measures to wild boar is challenging. Vaccinating could help, and it seems to be well accepted. Additionally, more biosecurity or control could help improve safety of wild boar product exchange.

## Data Availability

Upon reasonable request.

## Supplementary Materials

The following are available online at https://www.mdpi.com/article/10.3390/pathogens11020137/s1, Table S1: Domestic pig questionnaire agreed questions; Table S2: Wild boar questionnaire agreed questions.
