# Supplementary material for: African Swine Fever Survey in a European Context
Source: Pathogens. 2022 Jan 23;11(2):137. doi: 10.3390/pathogens11020137 (PMC8878522; doi:10.3390/pathogens11020137)
Supplement: Supplementary file 1 [file pathogens-11-00137-s001.zip › pathogens-1525394-supplementary.pdf]

SUPPLEMENTARY MATERIAL- EXPERT ELICITATION WILD BOAR AND DOMESTIC PIG RISK FACTORS FOR ASF CONTROL

Agreed questions in the domestic pig questionnaire (Table S1) and in the wild boar questionnaire (Table S2) with type of answer in brackets. The order in the table does not necessarily follow the order in the online survey questionnaire.

Table S1. Domestic pig questionnaire

**Pig farming questions**	
1	Occurrence of backyard pig farming (yes/no)
2	Frequency of backyard pig farming (>50% farms, <5% farms, <20% farms, <50% farms, other percentages)
3	Occurrence of swill feeding (yes/no)
4	Frequency of swill feeding occurrence in pig farms (not very common, somewhat common, quite common, very common)
5	Type of farm where swill feeding occurs (own consumption farms, small commercial farms, free-roaming but owned pigs- "village pigs"-)
6	Geographical areas where backyard farming is more predominant (free text)
7	Likelihood of home slaughtering pigs (very likely, likely, somewhat likely, somewhat unlikely, unlikely, not applicable)
8	Who performs home slaughters (farmer, slaughter man, both, nobody)
9	Likelihood of veterinary presence prior to home slaughtering (very likely, likely, somewhat likely, somewhat unlikely, unlikely)
10	Likelihood that pig owners also hunt wild boar (very likely, likely, somewhat likely, somewhat unlikely, unlikely)
11	Identification of pig and pig products movements (yes/no)
12	Frequency of moving pigs or pig products unidentified (<1% farmers, <20% farmers, <50% farmers, >50% farmers)
13	Geographical areas where unidentified movements are frequent (free text)
14	Where are illegal live pigs or products moved to (only locally, from and to other countries, they don't happen)
15	Likelihood of sharing live pigs as a gift (very likely, likely, somewhat likely, somewhat unlikely, unlikely)
16	Likelihood of sharing homemade pig products as a gift (very likely, likely, somewhat likely, somewhat unlikely, unlikely)
17	Season or event when exchange of pigs or pig products more likely (free text)
18	Do pig from different origins share or graze on communal land (yes/no)
19	Is it common to introduce pigs from multiple origins in farms (yes/no)
20	Frequency of multiple sources introduction or communal land sharing (<1% farmers, <20% farmers, <50% farmers, >50% farmers)
21	Geographical areas where introduction of pigs from multiple sources or communal land sharing is more common (free text)

**Wild boar-domestic pig potential interaction questions**	
1	Existence of hybrid pigs (yes/no/yes but…-free text-)
2	Likelihood of hybrid pigs existence (very likely, likely, somewhat likely, somewhat unlikely, unlikely)
3	Existence of free-range pigs as a pest, for own-consumption, local consumption or other (yes/no/yes, but…-free text)
4	Geographical areas where free-ranging or hybrid pigs are present

5	Environmental areas where free-ranging pigs are likely to interact with wild boar (based on Bosch et al, 2017 [39]: forest, shrubland, grassland, agroforestry, monoculture, urban close to natural areas, not likely) (more than one option possible)
6	Ranking of risk of direct contact between wild boar and domestic pigs (very high, high, medium, low, negligible, not applicable)
7	Access of wild boar to crops around farms (yes/no)
8	Frequency by season or whole year round of crop damage by wild boar (no damage, occasional, frequent)
9	Geographical areas where crop damage by wild boar is more frequent (free text)
10	Quantity of farms in suitable wild boar habitat (none or <1%; <20%, <50%; >60%; another quantity or range)
11	Frequency of manure or waste dumping in the field outside the farm (none or <1%; <20%, <50%; >60%; another quantity or range; it happens but cannot quantify)
12	Geographical areas where manure or waste dumping is more frequent (free text)
13	Frequency of collecting grass, straw, plants or other material from environment for farm use (none or <1%; <20%, <50%; >60%; another quantity or range; it happens but cannot quantify)
14	Geographical areas where collecting material from the environment for the farm is more frequent (free text)
15	Ranking of risk of indirect contact between wild boar and domestic pigs (very high, high, medium, low, negligible, not applicable)
16	Have *Ornithodoros* been searched for or found and how? (direct observation of tick, indirect observation of tick, both, none)
17	Location of *Ornithodoros* finding (not found, domestic pig farms, other livestock farms, hunted wild boar, dead wild boar, elsewhere in the environment)
18	Frequency by season for *Ornithodoros* findings if any? (occasional, very common, not common)
19	Mechanical vector species, frequency by season and species and geographical distribution (free text)

Probability and risk assessment questions

1	Introduction pathways (rank 1 to 7; 1= lower risk): legal trade of suids, illegal trade of suids, illegal trade of pork/wild boar meat and products, legal trade of pork/wild boar meat and products, catering waste, wild boar movements, own consumption meat
2	Exposure pathways (rank 1 to 8): wild boar direct contact, domestic pig direct contact, ticks, wild boar fomites, domestic pig fomites, feed waste, water sources, insemination
3	Perception on the main interaction between hosts association for ASF dispersal (more than one answer possible) (wild boar-wild boar; wild boar-domestic pig or vice versa; wild boar-tick-domestic pig; wild boar-human; domestic pig-human; domestic pig-domestic pig; domestic pig-tick; wild boar-tick; predators; scavengers)

ASF prevention and control, and impact of vaccination questions

1	Perception on stakeholders' awareness of ASF contingency planning (extremely aware, very aware, somehow aware, not so aware, not at all aware, there is no contingency plan for ASF or it is not updated)
2	Perception on commercial pig establishments' compliance with 12 control measures based on Jurado et al., 2018 [21] (<5%, <25%; <50%, >75% compliance)
3	Perception on non-commercial pig establishments' compliance with 10 control measures based on Jurado et al., 2018 [21] (<5%, <25%; <50%, >75% compliance)

4	How is active surveillance carried out (routine sampling on representative census-based design; sampling after epidemiological or clinical suspicion; sampling based on probability and consequences of being infected; other – free text –)
5	Who would likely pay for vaccination costs against ASF (more than one option possible) (government, swine industry, other, nobody)
6	Who would likely pay for vaccination costs against ASF in backyard or family pig establishments (more than one option possible) (government, swine industry, owners, other, nobody)
7	Who would likely pay for vaccination costs against ASF in commercial establishments (more than one option possible) (government, swine industry, other, nobody)
8	Perception on pig and pig products trade status (importer/exporter of live animals, importer/exporter of products)
9	Perception on likelihood of pig farmers' acceptance of ASF vaccination vs. current control strategies (very likely, likely, somewhat likely, somewhat unlikely, very unlikely)

Table S2. Wild boar questionnaire

Wild boar and hunting questions

1	How is wild boar considered in this country? (invasive, native, non-native species, based on OIE definition)
2	Is it common to hunt wild boar? (y/n)
3	Frequency by season or whole year round of wild boar hunting? (frequent, occasional, rarely for each season or whole year round)
4	What percentage of wild boar population constitute an average hunting bag of any given year? (<5%, 20-30%, >50%, free text for another percentage)
5	Hunters: professional or recreational? (both present in same proportion, more professional than recreational, more recreational than professional)
6	Distribution and abundance of wild boar (patchy or widespread combined with low or high abundance)
7	Population trend in the last 5 years (decrease, increased moderately, increased a lot, increased a great deal)
8	Environmental areas where wild boar is present (based on Bosch et al., 2017 [39]: forest, shrubland, grassland, agroforestry, monoculture, urban close to natural areas) (more than one option possible)
9	Frequency of poaching or illegal hunting (not common, somewhat common, very common)
10	Geographical areas where poaching is more common (free text)

Wild boar-domestic pig potential interaction questions

1	Main human activities carried out in wild boar habitats (list of options based on Petit et al., 2020 [46] but free text allowed for more options)
2	Frequency by season or whole year round of crop damage by wild boar (no damage, occasional, frequent)
3	Crops most commonly damaged by wild boar (free text)
4	Access of wild boar to urban areas or other areas with human garbage feeding on leftovers (not so common, somewhat common, very common, extremely common)
5	Have *Ornithodoros* ticks been searched for or found? (yes/no)
6	How have *Ornithodoros* been searched for or found? (direct observation of tick, indirect observation of tick, both, none)

7	Location of *Ornithodoros* finding (not found, domestic pig farms, other livestock farms, hunted wild boar, dead wild boar, elsewhere in the environment)
8	Frequency by season for *Ornithodoros* findings if any? (occasional, very common, not common)
9	Likelihood of contact, direct or indirect, between wild boar and domestic pigs (very likely, likely, somewhat likely, somewhat unlikely, unlikely)
10	Perception of most likely contact between wild boar and domestic pigs (direct contact, indirect contact, both)
11	Likelihood of hybrid pigs existence (very likely, likely, somewhat likely, somewhat unlikely, unlikely)
12	Geographical areas where hybrid pigs are present
13	Main wild boar predator species (free text)
14	Season in which predators would more likely feed on wild boar by species (free text)
15	Environmental areas in which predators would more likely feed on wild boar by species (free text)
16	Perception on role of predators in risk of ASF spread (reduction of risk; contribution to risk; no risk)
17	Main wild boar scavenger species other than suids (free text)
18	Season in which scavengers would more likely feed on wild boar by species (free text)
19	Environmental areas in which scavengers would more likely feed on wild boar by species (free text)
20	Perception on role of scavengers in risk of ASF spread (reduction of risk; contribution to risk; no risk)

Probability and risk assessment questions

1	Introduction pathways (rank 1 to 7; 1= lower risk): legal trade of suids, illegal trade of suids, illegal trade of pork/wild boar meat and products, legal trade of pork/wild boar meat and products, catering waste, wild boar movements, water sources
2	Probability of introduction/reintroduction of ASF from neighbouring countries through wild boar corridors or patches of shared habitat across borders? (extremely probable, somewhat probable, could happen but not so probable, negligible)
3	Exposure pathways (rank 1 to 7): wild boar direct contact, domestic pig direct contact, ticks, wild boar fomites, domestic pig fomites, feed waste, water sources

ASF prevention and control, and impact of ASF vaccination questions

1	General measures against ASF in wild boar (more than one option possible) (zoning and movement restrictions, PCR testing in dead wild boar, PCR testing in hunted wild boar, PCR and antibody testing in hunted wild boar, fencing, depopulation, selective hunting, other, none)
2	Are there specific biosecurity measures during carcass removal and disposal in the field? (yes/no)
3	Are rendering plants available for storage and destruction of infected wild boar cases in case there is ASF? (yes/no)
4	Supplementary feeding legality (legal, illegal, legal but with measures to reduce ASF transmission risk)
5	Frequency by season of supplementary feeding (very rare, frequent, N/A)
6	Geographical areas where supplementary feeding takes place (free text)
7	Existence of a shared surveillance programme for ASF in wild boar with neighbouring countries (yes/no)

8	Location of signs to prevent against the introduction or spread of ASF in the country (more than one answer possible)
9	Most difficult action to implement in wild boar to control ASF spread (free text)
10	Who would likely pay for wild boar vaccination against ASF (more than one option possible) (government, swine industry, hunters' associations, other, nobody)
11	Perception on likelihood of hunters' acceptance of ASF vaccination vs. current control strategies (very likely, likely, neither likely nor unlikely, unlikely, very unlikely)
12	Perception on wild boar trade status (importer/exporter of live animals, importer/exporter of products)
13	Perception on wild boar hunting tourism value (extremely valuable, very valuable, somewhat valuable, not so valuable, not at all valuable)
14	Legality of trade movement of live wild boar for hunting purposes (Legal in/out/within; Illegal in/out/within; not allowed)
15	Is it common to share or exchange wild boar products (yes-wild boar product; yes-hunting trophy; yes- both; no)
